# Nintedanib-Associated Generalized Body Swelling in a Patient With Idiopathic Pulmonary Fibrosis: A Case Report

**DOI:** 10.7759/cureus.115529

**Published:** 2026-08-31

**Authors:** Abdulaziz I Alghulayqah

**Affiliations:** 1 Department of Medicine, College of Medicine, Qassim University, Buraydah, SAU

**Keywords:** adverse drug reaction, drug rechallenge, generalized body swelling, generalized edema, idiopathic pulmonary fibrosis, interstitial lung disease, nintedanib, pharmacovigilance, tyrosine kinase inhibitor, usual interstitial pneumonia

## Abstract

Idiopathic pulmonary fibrosis is a chronic, progressive fibrosing interstitial lung disease with a poor prognosis, carrying a median survival of two to three years without antifibrotic therapy. Nintedanib, an oral intracellular tyrosine kinase inhibitor (TKI) targeting vascular endothelial growth factor receptors (VEGFRs 1-3), platelet-derived growth factor receptor (PDGFR)-α/β, and fibroblast growth factor receptors (FGFRs) 1-3, has emerged as a cornerstone of therapy, reducing the rate of forced vital capacity (FVC) decline by approximately 50% in the INPULSIS trials. While diarrhea is the most common adverse event, generalized body swelling has not been previously described with nintedanib, although it is a well-recognized class effect of several oncologic TKIs, including imatinib and mesenchymal-epithelial transition factor (MET) inhibitors. We report a 66-year-old male ex-smoker with a 45-pack-year smoking history and comorbidities including ischemic heart disease, bronchial asthma, and gastroesophageal reflux disease who presented with five months of progressive dyspnea, classified as modified Medical Research Council grade III. High-resolution computed tomography revealed bilateral peripheral reticulation with mild honeycombing, traction bronchiectasis, and basal predominance without ground-glass opacities, fulfilling the criteria for a definite usual interstitial pneumonia pattern. Spirometry demonstrated a moderate restrictive pattern (FVC, 65%), and autoimmune screening was comprehensively negative. Following multidisciplinary review, nintedanib was initiated at 100 mg twice daily and subsequently escalated to the standard 150 mg twice-daily dose. The patient developed mild generalized body swelling at initiation, which worsened markedly after dose escalation. Extensive cardiology, nephrology, endocrine, hepatic, and metabolic evaluations identified no alternative cause. Discontinuation of nintedanib resulted in near-complete resolution of swelling within two months. Rechallenge at a tertiary interstitial lung disease center precipitated recurrence of identical generalized swelling within one month, necessitating a second discontinuation. The temporal relationship, dose dependence, and reproducible recurrence on rechallenge strongly suggest a causal association between nintedanib and generalized body swelling in this patient. This previously unreported adverse event warrants clinician vigilance and formal pharmacovigilance reporting.

## Introduction

Idiopathic pulmonary fibrosis (IPF) is a chronic, progressive, fibrosing interstitial lung disease (ILD) of unknown etiology that primarily affects older adults. The condition is characterized histopathologically by a usual interstitial pneumonia pattern, with areas of fibroblast foci, honeycombing, and architectural distortion of the lung parenchyma [1]. IPF carries a poor prognosis, with a median survival of two to three years from the time of diagnosis in the absence of antifibrotic therapy [2]. A systematic review of 154 cohort studies and 16 randomized controlled trials reported pooled mortality proportions of 0.12 (95% confidence interval (CI) 0.09-0.14) at one to two years, 0.38 (95% CI 0.34-0.42) between two and five years, and 0.69 (95% CI 0.59-0.78) at ≥5 years in untreated patients, with a pooled mean overall survival of four years [3]. Consequently, early initiation of disease-modifying therapy is critical to slow the inexorable decline in lung function.

The diagnostic approach to IPF has been refined by international consensus guidelines. The 2011 American Thoracic Society (ATS), European Respiratory Society (ERS), Japanese Respiratory Society (JRS), and Latin American Thoracic Association (ALAT) evidence-based guidelines categorize high-resolution computed tomography (HRCT) findings into three diagnostic patterns: usual interstitial pneumonia (UIP) pattern (subpleural and basal predominance, reticular abnormality, honeycombing with or without traction bronchiectasis, and absence of features inconsistent with UIP), possible UIP pattern (subpleural and basal predominance, reticular abnormality, and absence of inconsistent features), and inconsistent with UIP pattern [1]. In the appropriate clinical context, a patient with unexplained dyspnea, bibasilar inspiratory crackles, and the typical demographic profile, a definitive UIP pattern on HRCT obviates the need for surgical lung biopsy [4].

For nearly a decade, the therapeutic landscape of IPF has been dominated by two antifibrotic agents: pirfenidone and nintedanib. Nintedanib is an oral, small-molecule intracellular tyrosine kinase inhibitor (TKI) that competitively and reversibly blocks the adenosine triphosphate binding pocket of three key receptor tyrosine kinases implicated in fibrogenesis: vascular endothelial growth factor receptors 1-3 (VEGFRs 1-3), platelet-derived growth factor receptors (PDGFRs) α and β, and fibroblast growth factor receptors (FGFRs) 1-3 [5]. By inhibiting these signaling cascades, nintedanib interferes with fundamental fibrotic processes, including fibroblast proliferation, migration, and differentiation, as well as the secretion of extracellular matrix proteins [6]. In human lung fibroblasts derived from patients with IPF, nintedanib has been shown to reduce PDGF- and FGF-mediated receptor phosphorylation, providing a mechanistic basis for its clinical efficacy [7].

The clinical efficacy of nintedanib was established in the pivotal INPULSIS program, comprising two parallel 52-week phase 3 trials (INPULSIS-1 and INPULSIS-2) that enrolled a total of 1,066 patients with IPF who were randomized 3:2 to nintedanib 150 mg twice daily or placebo [8]. The adjusted annual rate of FVC decline was significantly lower with nintedanib: -114.7 mL/year versus -239.9 mL/year in INPULSIS-1 (between-group difference, 125.3 mL; *P* < 0.001) and -113.6 mL/year versus -207.3 mL/year in INPULSIS-2 (between-group difference, 93.7 mL; *P* < 0.001) [9]. This represents approximately a 50% reduction in the rate of lung function decline, demonstrating a clinically meaningful slowing of disease progression [10]. More recently, the INBUILD trial extended the indication of nintedanib to patients with progressive fibrosing ILDs other than IPF [11].

The tolerability profile of nintedanib is well characterized. In the pooled INPULSIS analysis, the most frequent adverse events in the nintedanib group were diarrhea (reported in 62.4% of patients compared with 18.4% in the placebo group), nausea, nasopharyngitis, cough, vomiting, decreased appetite, bronchitis, and IPF progression [12]. Serious adverse events occurred at similar rates between nintedanib (30.4%) and placebo (30.0%) recipients; diarrhea led to permanent discontinuation of study medication in less than 5% of patients [8]. Elevated liver enzymes (alanine aminotransferase (ALT) or aspartate aminotransferase (AST) ≥ 3× the upper limit of normal) were uncommon but more frequent with nintedanib (approximately 5%) than with placebo (approximately 1%) in the IPF trials, with higher rates observed in patients with progressive fibrosing ILD [13]. Despite its shared mechanistic class with oncologic TKIs, major antiangiogenic class effects such as hypertension, arterial thromboembolism, bleeding, and gastrointestinal perforation have remained relatively infrequent with nintedanib in IPF trials [10].

Generalized body swelling (generalized edema) is a well-documented adverse effect of several TKIs in oncology, particularly imatinib (where periorbital edema is a hallmark) and small-molecule mesenchymal-epithelial transition (MET) inhibitors such as crizotinib and capmatinib (where peripheral edema is common) [14]. The mechanisms proposed for TKI-related edema include perturbation of VEGF/VEGFR signaling and consequent disruption of endothelial permeability, PDGFR-mediated effects on dermal dendrocytes and interstitial fluid homeostasis, and, in the case of MET inhibitors, disruption of hepatocyte growth factor/MET signaling that regulates vascular endothelial permeability [15]. Histopathological analysis of eyelid tissue from a patient with severe imatinib-induced periorbital edema revealed dermal dendrocytes expressing PDGFR and c-KIT, implicating these perivascular cells in the pathogenesis of the edema [16]. VEGFR2 inhibition and downstream Src-mediated phosphorylation of VE-cadherin have been shown to compromise adherens junction integrity and promote vascular leakage in preclinical models [17]. Clinical experience with VEGFR-targeting agents such as sunitinib and sorafenib has confirmed that perturbation of the VEGF pathway is associated with hypertension, proteinuria, hand-foot skin reaction, and tissue-level fluid accumulation [18].

To date, however, generalized body swelling has not been previously reported as an adverse event of nintedanib in patients with fibrosing lung disease. This report describes a patient with IPF who developed recurrent and reproducible generalized edema on two separate occasions following nintedanib initiation, with resolution upon drug discontinuation.

## Case presentation

A 66-year-old male with a known history of ischemic heart disease, bronchial asthma, and gastroesophageal reflux disease presented with progressive shortness of breath for five months. His dyspnea was classified as modified Medical Research Council dyspnea scale grade III. He was an ex-smoker with a 45-pack-year smoking history and had stopped smoking five months before presentation. High-resolution computed tomography of the chest showed bilateral peripheral reticulation predominantly involving the lower lobes, associated with mild honeycombing and minimal traction bronchiectasis. No ground-glass opacities were observed. Air trapping was also noted, particularly in both lower lobes. Overall, the radiological findings were suggestive of IPF (Figure 1).

**Figure 1:**
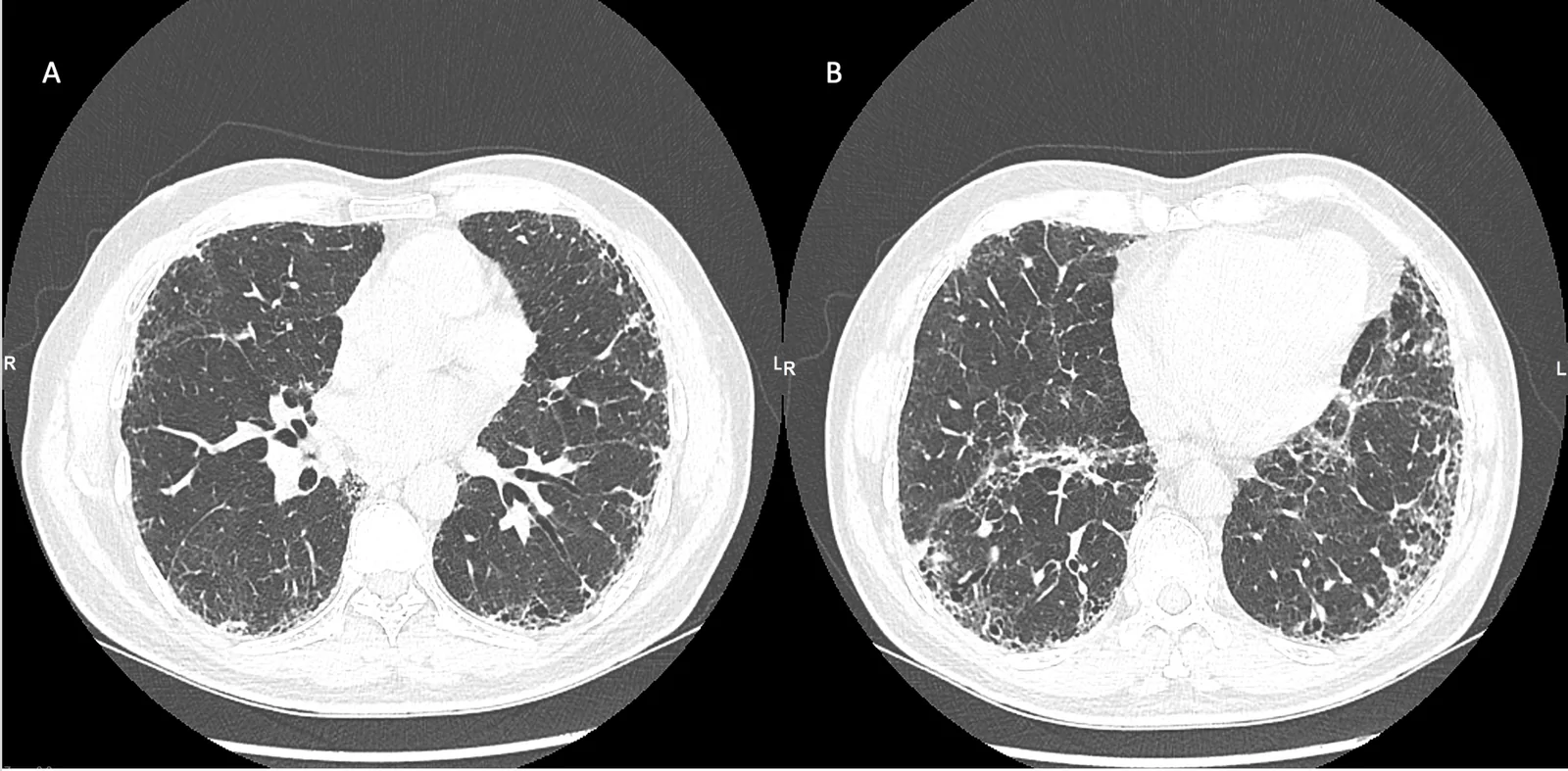
Chest CT demonstrating bilateral, subpleural-predominant fibrotic interstitial changes. (A) Bilateral subpleural reticulations. (B) More advanced fibrotic changes with bilateral subpleural reticulations, traction bronchiectasis, and focal areas of honeycombing, findings suggestive of a usual interstitial pneumonia (UIP) pattern.

Spirometry demonstrated a moderate restrictive pattern, with an FVC of 65%. Autoimmune screening was negative, including antinuclear antibody, anti-double-stranded DNA, anti-Jo-1, anticentromere antibody, anti-LA, anti-Ro, anti-cyclic citrullinated peptide, and rheumatoid factor. Echocardiography showed a normal ejection fraction and normal pulmonary artery systolic pressure. His respiratory symptoms showed mild improvement after optimizing bronchial asthma treatment, including budesonide-formoterol and tiotropium. The case was subsequently discussed in a multidisciplinary meeting, and initiation of antifibrotic therapy with nintedanib was recommended based on the clinical status and the typical high-resolution chest CT findings. Chest CT demonstrated bilateral, predominantly peripheral and subpleural fibrotic interstitial lung changes. Bilateral subpleural reticulations were evident, reflecting interstitial thickening and fibrotic remodeling predominantly involving the peripheral lung fields. The fibrotic changes were more pronounced, with bilateral subpleural reticulations accompanied by traction bronchiectasis and focal areas of honeycombing (Figure 1).

Nintedanib was initially started at a dose of 100 mg twice daily. After initiation, the patient began to notice mild, generalized body swelling, with no other significant adverse effects reported. During the second month of treatment, the dose was increased to 150 mg twice daily. At the third follow-up visit, he complained of significant generalized swelling, which was more pronounced over the face and limbs. A comprehensive evaluation was performed to identify the cause of the generalized swelling. Investigations included liver enzymes, complete renal function tests, urinalysis, 24-hour urinary protein collection, serum albumin, urine protein/creatinine ratio, serum calcium, thyroid function tests, pro-brain natriuretic peptide, and cortisol levels. All results were within normal limits. Nephrology and cardiology consultations were also obtained, but no specific underlying cause for the generalized swelling was identified. Detailed laboratory findings of the patient are summarized in Table 1.

**Table 1:** Baseline laboratory investigations of the patient. HPF, high-power field

Parameter	Patient value	Unit	Reference range
Complete blood count			
White blood cells (WBC)	9.47	×10⁹/L	4-11
Neutrophils	47.9	%	37-80
Neutrophils, absolute	4.54	×10⁹/L	2-6.9
Lymphocytes	39.8	%	10-50
Lymphocytes, absolute	3.77	×10⁹/L	0.6-4
Basophils	1.06	%	0-2.5
Basophils, absolute	0.1	×10⁹/L	<0.2
Eosinophils	1.32	%	0-5
Eosinophils, absolute	0.125	×10⁹/L	<0.5
Monocytes	9.8	%	1-11
Monocytes, absolute	0.91	×10⁹/L	0.2-0.9
Hemoglobin	15.4	g/dL	13-17.4
Hematocrit	42.5	%	39-52
Mean corpuscular hemoglobin (MCH)	32.0	pg	27-32
Mean corpuscular hemoglobin concentration (MCHC)	36.2	g/dL	29-37
Mean corpuscular volume (MCV)	90.1	fL	78-96
Mean platelet volume (MPV)	6.36 (decreased)	fL	7.4-10.4
Platelets	219	×10⁹/L	150-450
Red blood cells (RBCs)	4.81	×10¹²/L	4.04-6.13
Red cell distribution width (RDW)	11.2 (decreased)	%	11.6-15.5
Inflammatory markers			
C-reactive protein (CRP)	1.5	mg/L	<5
Erythrocyte sedimentation rate (ESR)	8	mm/hour	<15
Liver/Hepatobiliary profile			
Alkaline phosphatase (ALP)	44	U/L	40-150
Alanine aminotransferase (ALT)	24	U/L	5-55
Aspartate aminotransferase (AST)	18	U/L	5-34
Gamma-glutamyl transferase (GGT)	17	U/L	12-64
Direct bilirubin	2.5	µmol/L	<8.6
Total bilirubin	5	µmol/L	3.4-20.5
Albumin	42.5	g/L	32-46
Renal function and electrolytes			
Creatinine	86.2	µmol/L	64-104
Urea	5.40	mmol/L	2.9-8.2
Sodium	140	mmol/L	136-145
Potassium	4.1	mmol/L	3.5-5.1
Corrected calcium	2.1 (decreased)	mmol/L	2.2-2.6
Endocrine/vitamin profile			
Thyroid-stimulating hormone (TSH)	1.850	mIU/L	0.3-4.2
Free thyroxine (free T4)	14.50	pmol/L	9-19
Cortisol (AM)	243	Nmol/L	171-536
Vitamin B12	316.00	pmol/L	141-489
25-Hydroxyvitamin D (25(OH)D)	62.70	nmol/L	50-250
Cardiac marker			
NT-proBNP	29.40	pg/mL	<125
Immunology/Rheumatology			
Rheumatoid factor (RF)	<1.0	IU/mL	<30
Anti-CCP antibodies	0.50	U/mL	0-5
Antinuclear antibodies (ANA)	10.54	U/mL	<20
Anti-double-stranded DNA	18.37	IU/mL	<201
Anticentromere antibody	3.15	U/mL	<20
Anti Jo-1 antibody	2.00	U/mL	<20
Anti-Ro (SS-A) antibody	2.00	U/mL	<20
Anti-LA (SS-B) antibody	2.00	U/mL	<20
Urine/Renal studies			
Random urine creatinine	10.30	mmol/L	2.48-19.2
Urine osmolality	625	mOsm/kg	50-1,400
Urine protein/creatinine ratio	0.06	mg/mg	<0.2
Epithelial cells	Nil	cells/HPF	Nil
Mucus	2+	Semiquantitative	Nil
Specific gravity	1.020	Unitless	1.005-1.030
Bacteria	Nil	/HPF	Nil
Bilirubin	Nil	Qualitative	Nil
Blood	Nil	Qualitative	Nil
Appearance	Clear	Qualitative	-
Color	Light yellow	Qualitative	-
Ketones	Nil	Qualitative	Nil
Leukocytes	Nil	Qualitative	Nil
Nitrites	Negative	Qualitative	Nil
Urine pH	5.5	Unitless	4.6-8.0
Protein	Nil	Qualitative	Nil
RBC	0-2	cells/HPF	Nil
Urobilinogen	Normal	Qualitative	Normal
WBC	0-2	cells/HPF	Nil
Urine crystals	Nil	/HPF	Nil
Glucose	Nil	Qualitative	Nil
Ammonium biurate crystals	Nil	/HPF	Nil
Amorphous phosphate crystals	Nil	/HPF	Nil
Glycemic marker			
Glycated hemoglobin (HbA1c)	5.49	%	<5.7

After discussion with the patient and his family, nintedanib was discontinued. Two months later, the patient returned with almost complete resolution of the generalized swelling. His family sought another opinion and took him to a tertiary center for follow-up in an interstitial lung disease clinic. Nintedanib was restarted at the tertiary center; however, after one month, the patient again developed generalized body swelling similar to the previous episode. The medication was subsequently stopped again. The temporal relationship between nintedanib initiation and the development of generalized swelling, improvement after discontinuation, and recurrence after rechallenge suggests a possible association between nintedanib and generalized edema in this patient. Causality was further assessed using the Naranjo Adverse Drug Reaction Probability Scale [19]. The patient achieved a total score of 8, indicating a probable causal relationship between nintedanib and generalized edema (Table 2).

**Table 2:** Naranjo Adverse Drug Reaction Probability Scale. Probability total score: ≥9, definite; 5-8, probable; 1-4, possible; 0, doubtful.

S. no.	Questions	Yes	No	Do Not Know	Score
1	Are there previous conclusive reports on this reaction?	+1	0	0	0
2	Did the adverse event appear after the suspected drug was administered?	+2	-1	0	+2
3	Did the adverse reaction improve when the drug was discontinued, or a specific antagonist was administered?	+1	0	0	+1
4	Did the adverse reaction reappear when the drug was readministered?	+2	-1	0	+2
5	Are there alternative causes (other than the drug) that could on their own have caused the reaction?	-1	+2	0	+2
6	Did the reaction reappear when a placebo was given?	-1	+1	0	0
7	Was the drug detected in the blood (or other fluid) in toxic concentrations?	+1	0	0	0
8	Was the reaction more severe when the dose was increased, or less severe when the dose was decreased?	+1	0	0	+1
9	Did the patient have a similar reaction to the same or similar drugs in any previous exposure?	+1	0	0	0
10	Was the adverse event confirmed by any objective evidence?	+1	0	0	0
	Total score				+8

The score was supported by the temporal relationship between drug exposure and symptom onset, improvement following drug withdrawal, recurrence upon rechallenge, worsening following dose escalation, and the absence of an identifiable alternative cause [19].

## Discussion

This case describes a previously unreported and reproducible association between nintedanib and generalized body swelling in an elderly male with IPF. Multiple mechanisms have been reported for generalized body swelling as a direct or indirect adverse effect of TKIs, particularly those used in oncology. However, to the best of our knowledge, generalized body swelling associated with nintedanib has not been previously reported. The temporal pattern, which is the onset of mild swelling at subtherapeutic dosing (100 mg twice daily), marked worsening after dose escalation to the standard 150 mg twice daily regimen, near-complete resolution after drug discontinuation, and rapid recurrence upon re-challenge at a tertiary center, strongly supports a causal, dose-related drug reaction.

The clinical differential diagnosis of generalized edema in an elderly patient with multiple comorbidities is broad. In this patient, cardiac causes were excluded by normal left ventricular ejection fraction and pulmonary artery systolic pressure on echocardiography and a normal pro-brain natriuretic peptide level. Renal and hepatic causes were excluded by normal renal function tests, liver enzymes, serum albumin, urinalysis, 24-hour urinary protein collection, and urine protein/creatinine ratio. Endocrine causes such as hypothyroidism and adrenal insufficiency were excluded by normal thyroid function tests and cortisol levels. Hypocalcemia and nephrotic-range proteinuria, which would have suggested alternative diagnoses, were also absent. The negative autoimmune panel, in conjunction with the characteristic HRCT findings of bilateral peripheral reticular abnormality, mild honeycombing, traction bronchiectasis, and basal predominance without ground-glass opacities, was consistent with a UIP pattern fulfilling the 2011 ATS/ERS/JRS/ALAT criteria for IPF [1] and supported the appropriateness of antifibrotic therapy.

Although nintedanib has not been officially associated with edema in published trials to date, edema is a recognized adverse effect of the broader TKI class. In oncology, the prototypical TKI-induced edema is imatinib-associated periorbital edema, which occurs in a substantial proportion of patients with chronic myeloid leukemia and gastrointestinal stromal tumors [20]. Histopathological studies of affected tissue have demonstrated an enrichment of PDGFR- and c-KIT-expressing dermal dendrocytes around dermal vessels, supporting a direct role for PDGFR inhibition in altering dermal interstitial fluid homeostasis [16]. Similarly, peripheral edema has been documented in up to a clinically significant proportion of patients receiving small-molecule MET inhibitors (crizotinib, capmatinib, tepotinib), and proposed mechanisms include perturbation of hepatocyte growth factor/MET signaling on vascular endothelium with consequent disruption of permeability balance and effects on renal sodium and water handling [15]. VEGFR inhibition is a third established pathway to edema: anti-VEGF agents such as bevacizumab, sorafenib, and sunitinib consistently produce hypertension, proteinuria, and tissue-level fluid retention, and VEGFR2-dependent Src-mediated phosphorylation of VE-cadherin has been shown experimentally to compromise adherens junction integrity and promote microvascular leakage [17,18]. Notably, sunitinib treatment in cancer patients has been shown to cause sustained cutaneous sodium accumulation, providing direct evidence that inhibition of the VEGFR pathway alters tissue-level fluid and electrolyte balance [21].

Nintedanib inhibits VEGFR (1-3), PDGFR (α and β), and FGFR (1-3) at clinically relevant concentrations [5,6]. Although the IPF dose of 150 mg twice daily is lower than the oncologic doses of TKIs that produce overt edema, two considerations make an on-target pharmacologic basis for the observed edema plausible. First, despite the lower absolute dose, nintedanib produces demonstrable class effects on VEGF-pathway biomarkers in patients, including elevations in blood pressure and rare thromboembolic and bleeding events consistent with VEGFR inhibition [10]. Second, interindividual variability in drug exposure due to age, body size, hepatic metabolism, and concomitant medications can produce functionally higher exposures in some patients; a small body surface area or low body mass index has been shown to predict nintedanib-related hepatotoxicity, supporting the principle that exposure-response relationships extend well beyond the median [22]. In this patient, mild edema developed even with the lower dose of 100 mg twice daily, suggesting heightened individual susceptibility rather than a simple dose-dependent effect, and the symptoms worsened upon dose escalation, consistent with a dose-response relationship.

It is also worth considering that older patients such as ours commonly have subclinical impairments in lymphatic and venous return, decreased albumin reserve, and altered capillary permeability, which may unmask or amplify drug-induced changes in interstitial fluid balance. The patient's pre-existing ischemic heart disease, despite preserved ejection fraction, could have contributed to a reduced reserve in venous return or capillary hydrostatic regulation.

In the INPULSIS trials, diarrhea was the only adverse event leading to permanent discontinuation in over 5% of patients, and cardiac, renal, hepatic, and thromboembolic events were carefully monitored without revealing a signal of generalized edema [8,10]. To the best of our knowledge, however, no formal post-marketing surveillance analysis to date has identified generalized body swelling as a labeled adverse event of nintedanib. Our patient's reproducible recurrence on re-challenge represents the strongest possible level of clinical evidence for an adverse drug reaction outside of a formal randomized re-exposure design. It highlights the importance of clinician vigilance and pharmacovigilance reporting for rare or unlisted adverse events.

The management challenge in this patient was substantial. Both pirfenidone and nintedanib are guideline-recommended antifibrotic therapies for IPF, and there is limited evidence-based guidance for switching or combining agents when a patient cannot tolerate one due to a rare adverse event [12]. Outside of the clinical trial setting, the patient's options included a trial of pirfenidone with monitoring, supportive care alone with serial monitoring of lung function, or, where available and appropriate, referral for lung transplantation evaluation, given that lung transplantation has been shown to substantially improve survival in selected patients with IPF [23].

## Conclusions

We report a case of reproducible, dose-related generalized body swelling associated with nintedanib therapy in a 66-year-old man with IPF confirmed by a typical UIP pattern on HRCT. The temporal relationship, dose dependence, recurrence on rechallenge, and absence of any alternative cause after comprehensive evaluation support a probable causal relationship. Although generalized edema is a recognized adverse effect of several other TKIs, including imatinib, MET inhibitors, and VEGFR-targeting agents, nintedanib has not previously been implicated. Given its mechanism as a multi-receptor TKI targeting VEGFR, PDGFR, and FGFR, and the established role of VEGF-pathway inhibition in altering endothelial permeability and tissue fluid balance, an on-target pharmacologic mechanism is biologically plausible. This case highlights the importance of post-marketing pharmacovigilance for rare adverse events. It suggests that generalized body swelling should be recognized as a potential, albeit uncommon, adverse effect of nintedanib in susceptible patients with IPF. Clinicians should maintain a high index of suspicion for drug-induced edema when unexplained body swelling arises during nintedanib therapy and consider temporal interruption followed by re-evaluation as both a diagnostic and management strategy.
